# A new structural insight into XPA–DNA interactions

**DOI:** 10.1042/BSR20140158

**Published:** 2014-12-12

**Authors:** Benjamin Hilton, Nick Shkriabai, Phillip R. Musich, Mamuka Kvaratskhelia, Steven Shell, Yue Zou

**Affiliations:** *Department of Biomedical Sciences, East Tennessee State University, J.H Quillen College of Medicine, Johnson City, TN 37614, U.S.A.; †Ohio State University Health Sciences Center, College of Pharmacy, Center for Retrovirus Research and Comprehensive Cancer Center, Columbus, OH 43210, U.S.A.; ‡Department of Biochemistry, Center for Structural Biology, 5140 Biological Sciences/MRB III, Vanderbilt University, Nashville, TN 37232, U.S.A.

**Keywords:** DNA-binding domain, DNA junction, nucleotide excision repair, XPA, XPA–DNA binding, DBD, DNA-binding domain, ds/ssDNA, double-strand/single-strand DNA, DTT, dithiothreitol, GGR, global genome repair, HGPS, Hutchinson–Gilford progeria syndrome, MALDI-TOF, matrix-assisted laser desorption time-of-flight, NER, nucleotide excision repair, NHS-biotin, N-hydroxysuccinimidobiotin, Q-TOF, quadrupole time-of-flight, RPA, replication protein A, TCR, transcription-coupled repair, XPA, *Xeroderma pigmentosum* group A

## Abstract

XPA (*xeroderma pigmentosum* group A) protein is an essential factor for NER (nucleotide excision repair) which is believed to be involved in DNA damage recognition/verification, NER factor recruiting and stabilization of repair intermediates. Past studies on the structure of XPA have focused primarily on XPA interaction with damaged DNA. However, how XPA interacts with other DNA structures remains unknown though recent evidence suggest that these structures could be important for its roles in both NER and non-NER activities. Previously, we reported that XPA recognizes undamaged DNA ds/ssDNA (double-strand/single-strandDNA) junctions with a binding affinity much higher than its ability to bind bulky DNA damage. To understand how this interaction occurs biochemically we implemented a structural determination of the interaction using a MS-based protein footprinting method and limited proteolysis. By monitoring surface accessibility of XPA lysines to NHS-biotin modification in the free protein and the DNA junction-bound complex we show that XPA physically interacts with the DNA junctions via two lysines, K168 and K179, located in the previously known XPA(98–219) DBD (DNA-binding domain). Importantly, we also uncovered new lysine residues, outside of the known DBD, involved in the binding. We found that residues K221, K222, K224 and K236 in the C-terminal domain are involved in DNA binding. Limited proteolysis analysis of XPA–DNA interactions further confirmed this observation. Structural modelling with these data suggests a clamp-like DBD for the XPA binding to ds/ssDNA junctions. Our results provide a novel structure-function view of XPA–DNA junction interactions.

## INTRODUCTION

Human XPA (*xeroderma pigmentosum* group A) protein plays a critical role in both GGR (global genome repair) and TCR (transcription-coupled repair) subpathways of NER (nucleotide excision repair) [1–7]. The importance of XPA in NER is illuminated by evidence demonstrating that of all XP phenotypes, patients deficient in XPA are most severely affected, XPA-deficient mice show a complete deficiency in NER, and XPA-deficient cells are most sensitive to UV irradiation [8–11]. Also importantly, a growing body of evidence links XPA to non-NER cellular processes. XPA has been showed to be involved in the mechanism of HGPS (Hutchinson-Gilford progeria syndrome) by binding to aberrant DNA replication forks in HGPS cells [12]; a recent finding suggests that XPA deficiency may lead to mitochondrial dysfunction [13].

XPA has long been thought to participate in DNA damage recognition in NER, working in cooperation with RPA (replication protein A) and the XPC–HR23B complex [2,4,5]. In the GGR pathway the XPC–HR23B complex is believed to be the factor that initially recognizes damage. In the TCR pathway, the XPC–HR23B complex is not required and RNA polymerase is believed to initiate recognition, leaving the role of XPA unclear [14]. The involvement of XPA in both subpathways of NER and it being a limiting factor for NER and UV damage repair illustrate the importance of XPA in the NER machinery [15]. Taken together, past studies imply that in addition to its DNA damage recognition role, XPA may have additional biochemical activities in NER and non-NER pathways.

Our previous work reported a potentially novel function of XPA in NER. We demonstrated that XPA has a much higher affinity for ds–ssDNA junctions with 3′- and/or 5′-ssDNA overhangs (including Y-structure junction and the junctions with either a 3′- or 5′-ssDNA branch) than for damaged dsDNA [16]. These are the same structures found as intermediates in NER. Furthermore, XPA recruits and anchors other NER proteins, especially the XPF–ERCC1 complex to repair sites for 5′-incision of damaged DNA [17–19]. The recruitment of the XPF–ERRC1 by XPA occurs near the ds–ssDNA junction of the unwound DNA intermediate at the adduct [20]. Other studies confirm that XPA is found near ds–ssDNA junctions where formation of pre-incision complexes is initiated [21]. The high binding affinity for junction structures provides an explanation for involvement of XPA in both subpathways of NER and for XPA remaining on the repair complex through the late stages of NER [9,22].

Junction structures are also present in many other DNA metabolic processes including non-NER DNA repair, replication and recombination. This implies that XPA may have additional roles in other cellular processes. An example is its involvement in HGPS via XPA–junction binding [12]. Also XPA was recently shown to interact with PCNA (proliferating-cell nuclear antigen) through its APIM domain [23]. These authors concluded that XPA is associated with the replisome in cells and is loaded onto newly synthesized DNA in undamaged cells. Therefore, XPA–junction interaction could play a role in this function. In addition, it was reported previously that XPA is able to bind double-stranded three-way or four-way Holliday junctions which were used to mimic the helical kinks induced by DNA damage [24,25]. Together, these studies demonstrate the important role of XPA–DNA junction interactions in DNA metabolism. Nevertheless, the structural aspects of the XPA–junction binding have yet to be determined.

XPA contains three main domains: an RPA-interacting domain, a TFIIH-interacting domain and a central or DBD (DNA-binding domain) [26,27]. The solution structures of the DBD of XPA were determined by NMR [26–28]. The interaction of XPA and its binding partners has been extensively studied; however, the structures of the full-length XPA–DNA complex remain to be defined.

In the present study, we conducted a structural determination of the binding of full-length XPA to DNA-junctions. By implementing a mass spectrometric protein footprinting method [29,30], specific protein–nucleic acid contacts of XPA–DNA interactions have been mapped. Then partial proteolysis coupled with protection through DNA binding [31] was performed and confirmed the identified contacts. We not only verify previously identified binding sites within the DBD, but also and importantly, reveal new DNA-binding sites outside the known DBD. Together our data shed light on the solution structures of the XPA–DNA complex with implication on the roles of XPA in NER and other non-NER cellular pathways.

## MATERIALS AND METHODS

### Protein purification and substrate labelling

Recombinant human XPA was expressed in insect cells and purified as previously described [32]. The protein concentration was determined by the Bradford assay (Bio-Rad). Oligonucleotides used to form the Y-shape, 5′-overhang and 3′-overhang substrates were synthesized by Integrated DNA Technologies, then radiolabelled with [γ-32P]ATP (>5000 mCi/mmol; PerkinElmer) using T4-PNK (Ambicon), and unincorporated nucleotides were removed by a P6 spin column (Bio-Rad). The labelled substrate then was annealed to a complementary strand, followed by gel purification in an 8% native polyacrylamide gel.

### Gel mobility shift assays

Effects of the NHS-biotin (N-hydroxysuccinimidobiotin) (Pierce) modification on XPA-junction binding were determined by gel mobility shift assays. The labelled Y-shape, 5′-overhang or 3′-overhang substrate (4 nM) was incubated with unmodified XPA in binding buffer [20 mM Hepes-KOH, pH 7.9, 75 mM KCl, 5 mM MgCl_2_, 1 mM DTT (dithiothreitol), 5% (v/v) glycerol and 1 mg ml^−1^ HSA] for 20 min at 30 °C. The preformed complex then was exposed to modification with increasing concentrations NHS-biotin for another 15 min. The amino acid lysine was added to 10 mM to quench the reaction. In parallel experiments, XPA was modified first with NHS-biotin, then junction DNA was added to the reaction mix. Reactions were immediately loaded onto 3.5% native PAGE and electrophoresed in TBE [Tris/borate/EDTA (45 mM Tris/borate and 1 mM EDTA)] buffer at 4 °C.

### Biotin/HPG modification and in-gel proteolysis

Purified XPA was modified with NHS-biotin in the presence and absence of each junction DNA. Typically, XPA (26.4 μM) was incubated with junction DNA (80 μM) in the binding buffer at room temperature for 30 min and then modified by adding NHS-biotin (500 μM final concentration) for an additional 30 min incubation at room temperature. Modifications were quenched by addition of 10 mM lysine, followed by separation of the XPA subunits by SDS–PAGE. The subunits were visualized by Coomassie Brilliant Blue staining, excised from the gel, and extensively destained in 50% (v/v) methanol/10% (v/v) acetic acid. SDS was removed by washing the gel pieces with ammonium bicarbonate, dehydrated with 100% acetonitrile and vacuum desiccated. Samples were digested with 1 μg of trypsin (Roche) in 50 mM ammonium bicarbonate overnight at room temperature. The supernatant then was recovered for MS analysis.

### MS analysis

MS spectra were obtained using MALDI-TOF (matrix-assisted laser desorption time-of-flight) and Q-TOF (quadrupole time-of-flight) techniques. MALDI-TOF experiments were performed using a Kratos Axima-CRF instrument (Kratos Analytical Instruments) with an R-cyano-4-hydroxycinnamic acid matrix. MS analyses were performed on a Micromass Q-TOF-II instrument equipped with an electrospray source and Micromass cap-LC. Peptides were separated with a Waters Symmetry 300 5 μm precolumn (Waters) and a Micro-Tech Scientific ZC-10-C18SBWX-150 column using two sequential gradients of 5–40% acetonitrile for 35 min and 40–90% acetonitrile for 10 min. MS sequence data and the MASCOT automated peptide search engine (www.matrixscience.com) were used to identify XPA peptide peaks from the NCBInr primary sequence database, and matched peaks were then located in the primary MS spectra. Protection events were qualitatively assigned as the appearance of a peak corresponding to a biotin-modified peptide in the modified protein spectrum and the absence of the modification peak in the modified nucleoprotein complex spectrum. A protection was considered to be significant only when the intensity of a modifiable peak was reduced by at least 85% in the nucleoprotein complex spectrum. To accurately identify protection events, at least two peaks that are not affected by procedures and present in all three spectra (unmodified protein, modified protein and modified nucleoprotein complex) were used as control peaks. These control peaks served to standardize the peak intensities in each spectrum for accurate qualitative assignment of protection. Data were reproducibly compiled and analysed from six independent experimental groups.

### Partial proteolysis and identification of proteolytic fragments

4 μg of recombinant human XPA was incubated with chymotrypsin (1:80::chymotrypsin:XPA) at 30 °C for the indicated times in 20 μl XPA binding buffer (20 mM Hepes-KOH, pH 7.9, 75 mM KCl, 5 mM MgCl_2_, 1 mM DTT, 5% glycerol and 1 mg/ml HSA). At each time point (5, 20 or 60 min), 5 μl of the reaction mixture was removed and terminated by the addition of Laemmli sample buffer and boiled for 5 min. The proteolytic products were resolved by SDS–PAGE (15% gel) and stained with SYPRO Ruby protein gel stain (Bio-Rad). After destaining with a solution containing 10% methanol and 7% acetic acid, the gel was imaged in a phosphorimager (FLA-5000, FUJIFILM) using a 473-nm laser line. For automated N-terminal sequencing, the proteolytic fragments separated on the SDS–PAGE (15% gel) were transferred to a polyvinylidene difluoride membrane followed by staining with Coomassie Brilliant Blue R-250. The protein bands of interest were excised and sent to the protein chemistry laboratory at Iowa State University for sequencing.

## RESULTS

### Effect of lysine modification on XPA–DNA interactions

**Figure 1:**
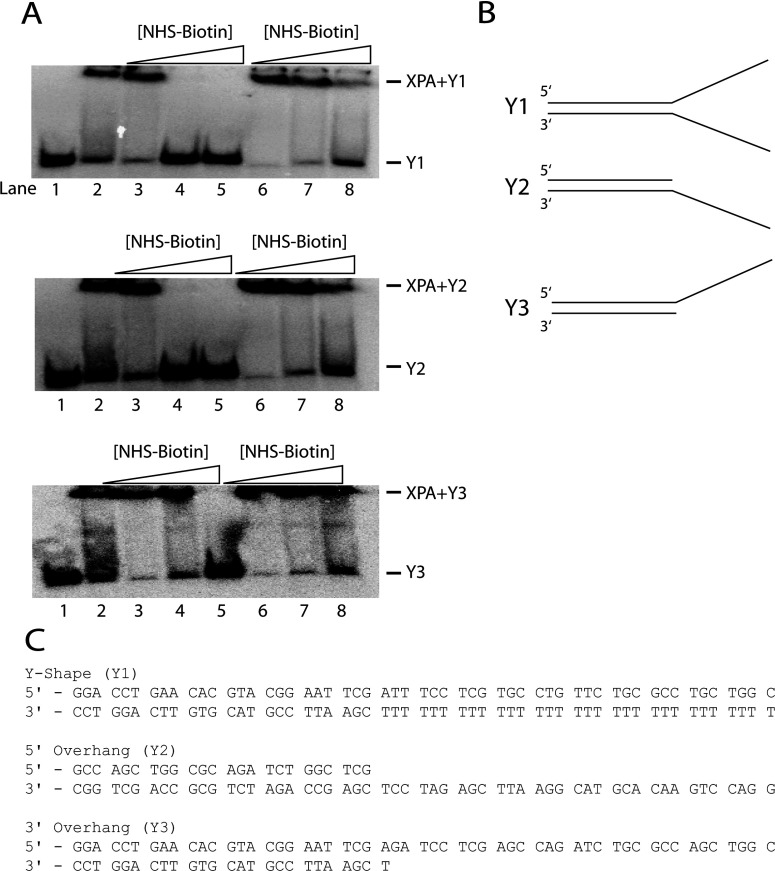
Effects of biotinylation on XPA–DNA junction binding (**A**): Lanes: 1, free junction DNA; 2, junction DNA–XPA complex; 3–5, junction DNA bound by XPA pretreated with 300, 500 and 800 μM NHS-biotin; 6–8, preformed XPA-junction DNA complex treated with 300, 500 and 800 μM NHS-biotin. Lanes 3–5 show that addition of increasing amounts of NHS-biotin prior to addition of junction DNA blocks critical lysine residues from junction DNA interaction, abolishing binding. Lanes 6–8 show formation of a XPA–junction DNA complex prior to adding NHS-biotin shielded critical lysine residues from subsequent modification and showed minor effects of biotin on the stability of the preformed XPA–junction DNA complex. Panel (**B**) shows substrate profiles used in all experiments: Y-shaped DNA (Y1); 5′-overhang DNA (Y2); 3′-overhang DNA (Y3). Panel (**C**) indicates the DNA sequence for each oligonucleotide.

We employed a protein footprinting [29,31–34] by which modification of solvent-accessible XPA lysine residues by the primary amine-specific reagent NHS-biotin is prevented at the XPA–DNA junction binding sites, while the surface lysines not involved in the binding are available for modification. NHS-biotin was used to insure minimal XPA–DNA junction complex disruption and maximum modification. And complex integrity and efficient modification by NHS-biotin must be confirmed. As shown in Figure 1(A), gel mobility shift assays were implemented to demonstrate the effect of increasing concentration of NHS-biotin on the binding of XPA to the DNA junction. XPA was incubated in the presence of NHS-biotin before or after addition of DNA junction substrates (lanes 3–5 and 6–8, respectively). The effect of modification on the binding was tested for three different types of DNA junction (Figure 1B). The data in Figure 1(A) showed that addition of increasing concentrations of NHS-biotin prior to nucleoprotein complex formation reduced and eventually eliminated the binding at 800 μM NHS-biotin (Figure 1A, lane 5) for all junction structures. This indicates that the lysine residues which can be modified by NHS-biotin are required for interaction of XPA with DNA junctions. Conversely, when NHS-biotin was added at increasing concentrations following nucleoprotein complex formation the binding was only slightly reduced (Figure 1A, lane 8). This indicates that the lysine residues involved in complex formation are shielded by the protein–DNA interactions. Based on these results a concentration of 500 μM NHS-biotin was chosen for the following studies as it is sufficient for modification of solvent-accessible lysine residues of XPA to prevent binding while having minimal effects on complex integrity. The three ds-ssDNA junction substrates (Figures 1B and 1C) yielded essentially identical results; therefore, all of the data presented hereafter are the representative data collected on substrate Y1. Also, it should be pointed out that as we reported previously XPA has little or no measurable affinity for either non-damaged dsDNA or ssDNA [16].

### MS analysis of the XPA–DNA complex

**Figure 2:**
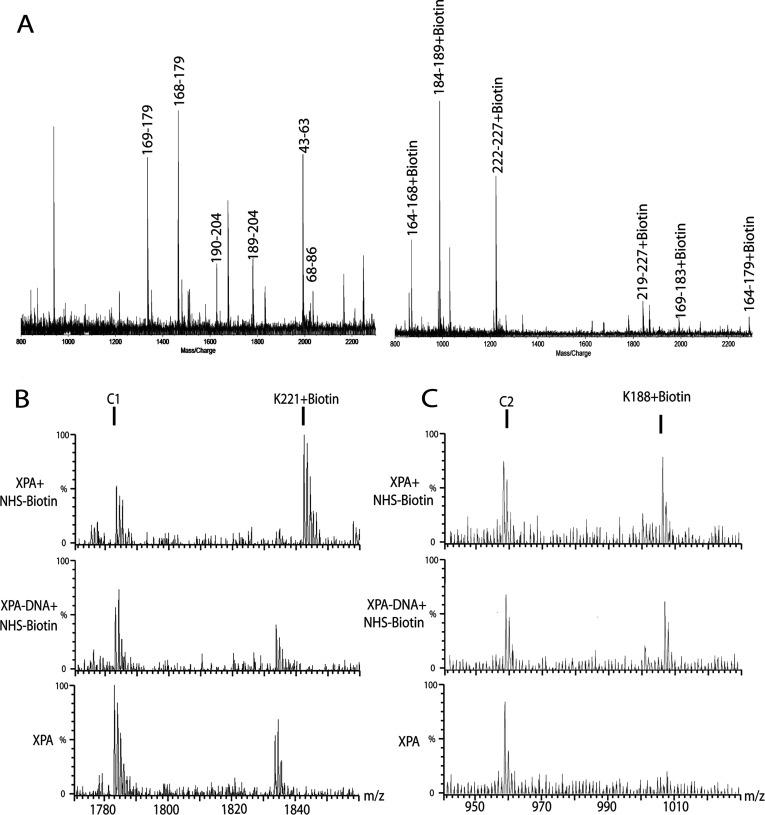
Identification of lysine residues involved in DNA binding (**A**) A typical MALDI-TOF spectrum of tryptic digests of XPA protein (left) or biotinylated XPA protein (right). Monoisotopic resolution for all of the peptide peaks were obtained, allowing unequivocal assignment of singly-charged unmodified and biotinylated peptide fragments. (**B**) the typical Q-TOF data illustrate the protection of a lysine residue in XPA. The peak corresponding to peptide fragment 221, 222, 224+biotin in XPA is an example of protection from modification by binding of ds–ssDNA junction DNA. When junction DNA is not present, K221, K222 and K224 is readily modified by NHS-biotin; however, in the presence of junction DNA the modification peak disappears. (**C**) in contrast, K188 is a lysine residue not protected by junction DNA binding. A modification peak appears upon treatment with NHS-biotin and persists following addition of junction DNA. Each multiply-charged peptide resulted in clearly resolved peak clusters, indicating monoisotopic resolution; unmodified peaks C1 and C2 serve as controls.

The NHS-biotin modified XPA, modified XPA–DNA junction complex, and unmodified XPA were subjected to SDS–PAGE prior to trypsin proteolysis. SDS–PAGE electrophoresis rendered the protein in a linear, denatured form that exposed all possible trypsin proteolytic sites, ensuring a complete and reproducible hydrolysis of the protein. The XPA lysines modified with NHS-biotin were detected by MS analysis of trypsin-generated peptide fragments. Figure 2(A) shows a representative MALDI-TOF spectrum for NHS-biotin-modified XPA with mass/charge peaks assigned to proteolysis-generated peptide fragments. This allowed for mono-isotopic resolution of the peaks reliably identifying the expected and biotin-modified tryptic fragments of XPA.

To focus on which lysines specifically interact with DNA in the nucleoprotein complex we analysed the mass spectrum for *m*/*z* peaks that are present in the modified-free protein but absent in both the modified nucleoprotein complex and the unmodified-free protein spectra. These peaks corresponded to the mass of a trypsin-generated fragment plus the mass of the bound NHS-biotin molecules. The number of NHS-biotin molecules attached to any tryptic peptide was equal to the number of missed lysine cleavage sites within the fragment. Figure 2(B) shows MALDI-TOF analysis with a typical assignment of lysine protection. When free XPA was NHS-biotin modified, a peak corresponding to XPA peptide fragment 219–227 (K221, 222, 224+biotin) was observed. However, when XPA–DNA complex was modified, this peak was absent, indicating shielding of lysine 221, 222 and 224 in the nucleoprotein complex from modification. This NHS-biotin modified peak also was absent in the untreated free XPA protein. In contrast, lysine K188, however, was not protected from NHS-biotin modification by XPA–DNA junction binding as the peak corresponding to peptide fragment 184–189 (K188+biotin) persists in both the free and DNA-bound XPA spectra (Figure 2C). Peaks C1 and C2 serve as internal controls to provide a reference for peak intensity for each spectrum.

**Figure 3:**
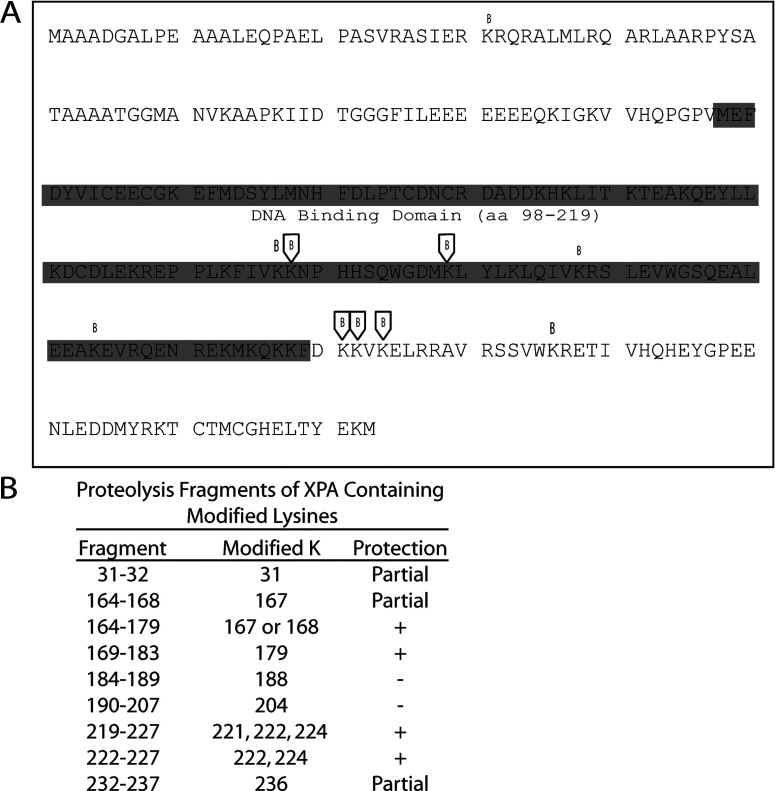
Summary of the footprinting results in the context of the XPA sequence (**A**) Biotinylation sites in XPA are indicated in the primary amino acid sequence either as protected (boxed B) or as unprotected residues (unboxed B). The location of the DBD is indicated by the shaded sequence plus the name and amino acid numbering of the structure. Panel (**B**) tryptic digest fragments of XPA are shown with the modified lysine residue indicated. Lysines that are shown to be protected from modification in the presence of ds–ssDNA junctions are indicated by (+) while residues readily modified in the presence and absence of ds–ssDNA junction are indicated by (−). Lysine residues that were only partially protected are indicated by (Partial). Even though the evidence was not as strong for ‘Partial’ residues, it is likely there is interaction of these lysines with ds–ssDNA junctions.

In our analysis, five lysine residues in XPA were found to be protected from NHS-biotin modification by DNA binding (Figure 3). Two of these lysines, K167/168 and K179, were previously shown to be located in DBD of XPA and thus can serve as an internal and positive control for the method of our footprinting analysis. Interestingly, the three lysines, K221, K222 and K224, have not been previously identified to be involved in XPA interaction with DNA. As summarized in Figure 3(B), two other lysines outside of the binding domain were partially protected, K31 and K236. These lysines also may be involved in DNA junction binding. These newly uncovered DNA-binding residues are of particular interest as they are located outside of the DBD (residues 98–219) and may play an important role in the structure-function relationship of XPA.

### Limited proteolysis of XPA–DNA junction complex

**Figure 4:**
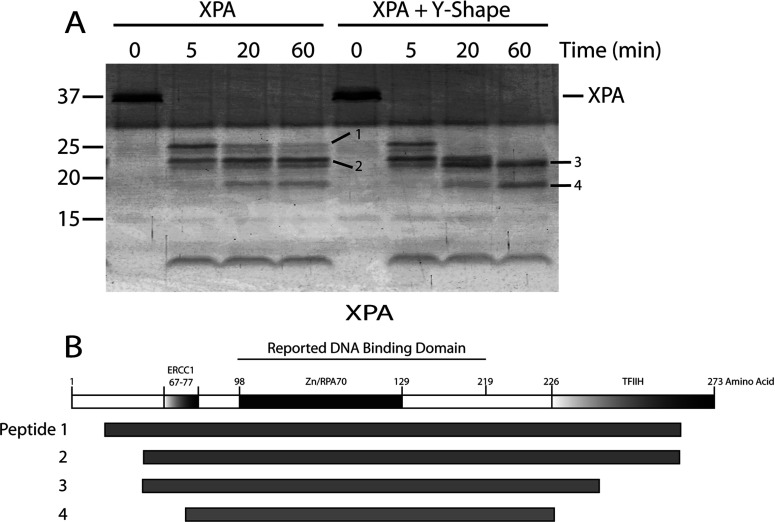
Partial proteolysis of XPA versus XPA–DNA junction complex by chymotrypsin (**A**) Four microgram of XPA was digested with chymotrypsin (1:80 molar ratio of chymotrypsin:XPA) at room temperature for the times indicated. The reactions were terminated and the cleavage products resolved by SDS–PAGE (15% gel) then visualized using SYPRO Ruby stain. Untreated XPA was loaded as a control (0 time). The molecular mass markers are indicated on the left. Individual fragments of interest are designated by dashes with numbers. (**B**) Schematic map of XPA with its functional domains highlighted followed by a schematic of the four proteolytic peptides indicated in (A) that were generated upon XPA treatment with chymotrypsin.

Partial proteolysis has been widely used to map a protein's domains [31,35–37]. Accessible proteolytic cleavage sites are usually located in loops between domains on the surface of a protein. Protein binding to DNA could change the accessibility of some of the cleavage sites, which would alter the proteolytic cleavage pattern [38]. In this study the cleavage pattern of XPA, in the presence or absence of DNA junction substrates, was analysed and compared to determine which regions interacted with DNA junction. XPA protein was partially digested before or after the addition of DNA junction with a fixed amount of chymotrypsin for the indicated time periods (Figure 4). In the case of XPA binding to junction DNA, several peptides were more sensitive while others were more resistant to cleavage as shown in Figure 4(A) (peptides 1 and 2 versus peptides 3 and 4, respectively). Three N-terminal cleavage sites of these peptides were determined by protein microsequencing. These chymotrypsin-sensitive sites occurred at amino acid positions 26, 48 and 75 in XPA (Figure 4B, Table 1). The calculated C-terminal cleavages occurred approximately at three amino acid positions 246, 238 and 226 for peptides 1 and 2, 3 and 4 (Table 1), respectively. The determined peptides were summarized in Figure 4(B). Upon XPA–DNA complex formation only peptides 3 and 4 persisted at 60 min of chymotrypsin digestion (Figure 4A). The shift from peptides 1 and 2 to peptides 3 and 4 suggests that XPA binding to junction DNA led to conformational changes, making peptide bonds at positions 48, 75, 226 and 238 significantly more sensitive to proteolytic cleavage. This was consistent with the NHS-biotin data that the novel DNA-binding site containing K221, K222 and K224 was protected from modification in the XPA–DNA junction complex. This region falls just outside the known DBD of XPA. These data could also suggest that K236, found to be partial protected, could also be involved in binding. Taken together, the partial proteolysis data confirmed the higher resolution results obtained from mass spectrometric footprinting.

**Table 1:** Identification of the peptides generated by chymotrypsin cleavage of XPA with and without bound junction DNAs

Peptide*	Size (kDa)^†^	N-terminal sequence	Location^‡^
1	26	SIER	27–246
2	23	SATA	49–246
3	22	SATA	49–238
4	18	ILEE	76–226

*Peptides are numbered as illustrated in Figure 4(A).

^†^Peptide sizes are estimated from their electrophoretic mobility relative to the Bio-Rad Precision Plus size standards.

^‡^The location of the peptide in the intact XPA sequence is calculated as the position of the first amino acid in the determined N-terminal sequence plus the number of amino acids corresponding to this size of the peptide as to the nearest likely chymotrypsin-cleavage site using Geneious Software.

## DISCUSSION

A growing body of recent studies has shown that XPA plays an important role not only in NER but also in some other DNA metabolic pathways, not necessarily related to DNA damage. This is consistent with the fact that XPA has a much higher affinity for DNA junctions than for damaged dsDNA [16]. The former is a structure commonly present as the intermediate in many DNA metabolic pathways, including NER. Given the importance of XPA, defining the structural aspects of XPA–DNA interactions is essential for understanding the mechanisms of XPA in DNA metabolism. The present study represents an effort to understand the structure–function of XPA by generating the structural information on the native full-length XPA interacting with DNA.

We have identified at least six lysine residues of XPA and the corresponding domain involved in the XPA–DNA junction interactions. Of them, K168 and K179 were located in the reported DBD of XPA and previously shown to be aligned with DNA in a modelling study [39]. It also has been showed previously that K179 interacted with damaged DNA in the absence of RPA70 leading to the question of whether the absence of RPA70 freed K179 of XPA to interact with DNA [40]. In contrast to K169 and K179 which serve nicely as internal and positive controls for this study, the newly identified lysine residues K221, K222, K224 and K236 are all located in the C-terminal domain relative to the known DBD. The C-terminal domain has not been shown previously to interact with DNA. Our results expand the DNA-binding regions of XPA from the previously known DBD to the C-terminal domain region, suggesting that redefining the DBD of XPA may be necessary.

**Figure 5:**
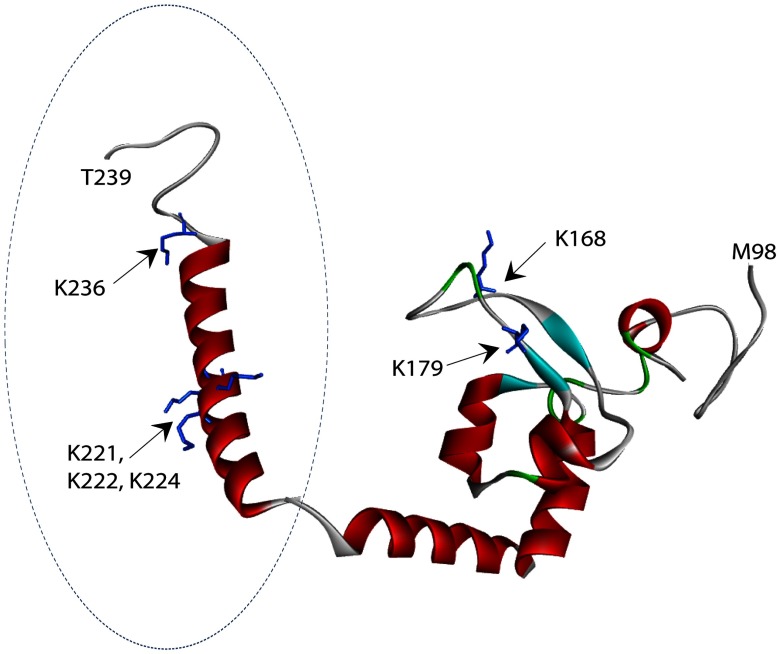
A structural model of redefined DBD of XPA Structure of the previously reported DBD (aa98-219) of XPA is shown with the predicted extended-structure generated by RaptorX (aa217–239, indicated by dashed circle). Structural mode of the redefined DBD of XPA was generated by Discovery Studio tethering of the two structures together by overlapping three amino acids shared between the two structures. Lysine residues in the structure are presented in stick representation. K168 and K179 are found in the previously reported DBD of XPA, while K221, K222, K224 and K236 are found within the extended structure. The two structures resemble two arms of a clamp. Biotin-modified lysine residues protected from modification in the presence of ds–ssDNA junctions are shown in blue.

To understand how the newly identified residues and domain are involved in XPA–DNA junction binding, a predicted structure of the C-terminal XPA217-239 was generated based on the sequence of XPA217-273 using RaptorX software package [41–43]. Discovery Studio software then was used to tether this predicted structure with the published solution structure of XPA DBD (pdb: 1XPA) by overlapping three amino acids shared between the two structures. Thus a structural model of XPA98–239 was generated (Figure 5). In this model, the newly identified K221, K222 and K224 are located in a α-helical structure with K221, K222 and K224 open to the binding cleft of the known DBD. In contrast, K168 and K179 are located in the β-sheets of the known DBD facing the C-terminal α-helix. Interestingly, this structural model is featured with two linked arms with the two groups of lysines located separately in each of the arms. The space between the two arms appears to form a perfect path for docking DNA. The C-terminal arm appears to be quite flexible so that it could accommodate DNA structures of varying size by interacting with the ss- and/or ds- regions. Thus the structure serves more like a clamp in light of DNA binding. This structure model also shows some similarity to the ‘right hand’ structure commonly found in many DNA polymerases. The two arms resemble the ‘thumb’ and ‘fingers’ while the region connecting the two arms resembles the ‘palm’ although it contains no catalytic activity found in polymerases.

To determine whether XPA could adopt different structures in binding different types of DNA junction, three different DNA junction substrates were used (Figure 1). However, we observed no significant difference in the structural determination of XPA binding to these substrates. A possible explanation for the results is that besides binding to the double-stranded region at the junction, XPA may additionally bind only to one ssDNA sequence without preference for either a 3′- or a 5′-ssDNA overhang. However, it is also possible that some structural differences may occur in non-lysine-containing regions of XPA but could be at the minimal level as our limited proteolysis analysis showed no differences among these substrates.

It is worth noting that the very recent work by Sugitani et al. analysed an extended DBD of XPA. Using a fluorescence spectroscopy method they demonstrate that residues outside of the established DBD are not only involved in binding but increase affinity by 5-fold using y-shaped DNA junctions [44]. Their NMR data confirms that the extended DBD binds to junction DNA much stronger than a construct with only the traditional shorter binding domain length. Their finding that XPA98–239 binds at a higher affinity to junction DNA as compared with duplex DNA shows not only that XPA's DBD is more extensive than previously thought but that XPA's main function in the cell could have been initially misinterpreted. These are in agreement with our findings in this study.

Finally, we realize that although our MS-based protein footprinting, limited proteolysis and structural modelling results all support the role of K221, K222, K224 residues and the C-terminal domain of XPA in XPA–DNA junction interactions, further confirmation would be expected. For example, the importance of this new finding could be strengthened in future mutagenesis studies to determine the involvement of each of these identified lysine residues in the XPA-junction interaction. In addition, it is of interest to define the minimum lengths of both dsDNA and ssDNA of DNA junction for efficient XPA binding.

## Online data

Supplementary data
