# Supplementary material for: A new structural insight into XPA–DNA interactions
Source: Biosci Rep. 2014 Dec 12;34(6):e00162. doi: 10.1042/BSR20140158 (PMC4266923; doi:10.1042/BSR20140158)
Supplement: Supplementary data [file bsr034e162ntsadd.pdf]

Figure S1

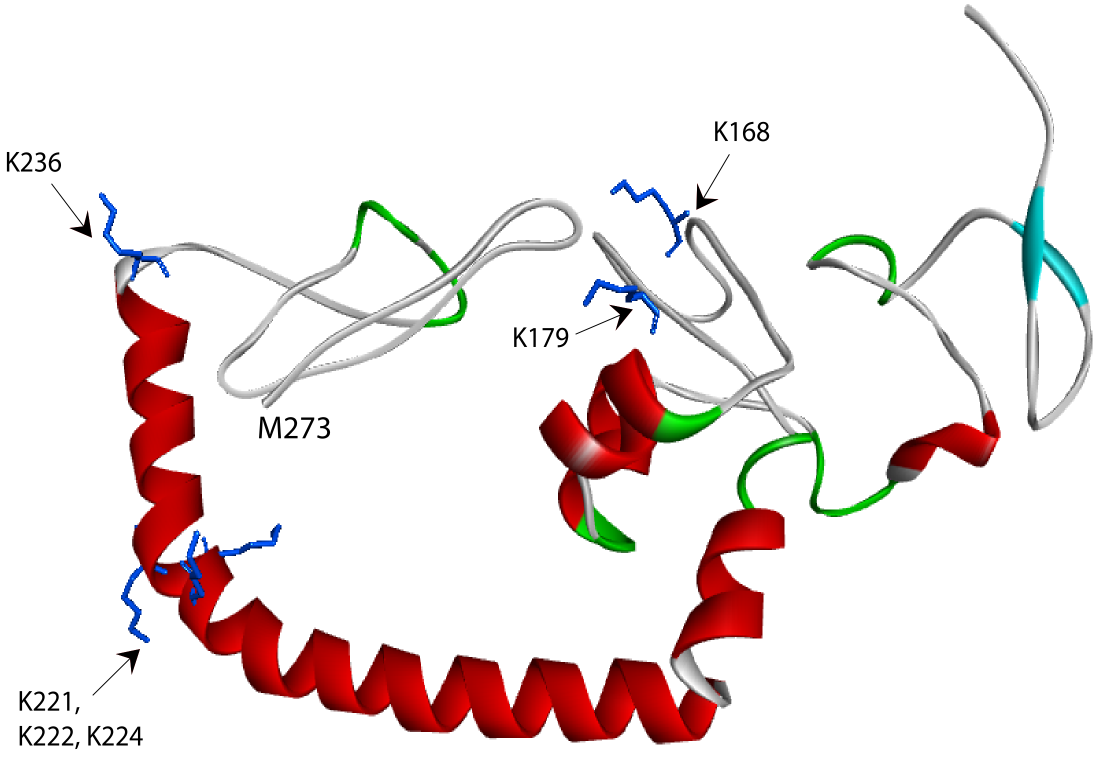

Figure S1. I-TASSER structural model of redefined DNA binding domain of XPA.
This representative model was generated by submitting the redefined XPA DNA binding domain amino acid sequence (aa98-273) for protein structure prediction to the I-TASSER software package. 5 models were generated and the model with best fit was selected. Lysine residues in the structure are presented in stick representation. K168 and K179 are found in the previously reported DNA-binding domain of XPA, while K221, K222, K224, and K236 are found within the extended structure. The two structures resemble two arms of a clamp. Biotin-modified lysine residues protected from modification in the presence of ds-ssDNA junctions are shown in blue.

Yang Zhang. I-TASSER server for protein 3D structure prediction. BMC Bioinformatics, 9:40 (2008).

Ambrish Roy, Alper Kucukural, Yang Zhang. I-TASSER: a unified platform for automated protein structure and function prediction. Nature Protocols, vol 5, 725-738 (2010).

Ambrish Roy, Jianyi Yang, Yang Zhang. COFACTOR: an accurate comparative algorithm for structure-based protein function annotation. Nucleic Acids Research, vol 40, W471-W477 (2012).
